# Characterization of Synthesized Ramucirumab-vcMMAE as a Potential Therapeutic Approach in Ovarian Cancer

**DOI:** 10.1021/acsomega.5c03733

**Published:** 2025-08-27

**Authors:** Duygu Erdogan, Hulya Ayar Kayali

**Affiliations:** † Izmir International Biomedicine and Genome Institute, 199479 Dokuz Eylül University, Izmir 35340, Türkiye; ‡ Izmir Biomedicine and Genome Center, Izmir 35340, Türkiye; § Department of Chemistry, Division of Biochemistry, Faculty of Science, Dokuz Eylül University, Izmir 35390, Türkiye

## Abstract

Current chemotherapy for ovarian cancer, often detected at a late stage, causes side effects and drug resistance. This highlights the necessity for targeted drug delivery systems. The study focuses on in vitro testing of Antibody-Drug Conjugate (ADC) for smarter, more selective cancer cell targeting. In the study, the conjugate was synthesized by reducing the interchain disulfide bonds of Ramucirumab, followed by alkylation with mc-vc-PAB-MMAE (vcMMAE). The synthesized conjugate underwent structural, physicochemical, and functional analyses, followed by an assessment of its in vitro efficacy in ovarian cancer cell lines with normal, primary and metastatic characteristics. It was found that Ramucirumab-mc-vc-PAB-MMAE (R-vcMMAE) had a mean drug-to-antibody ratio of 3.2 and a monomeric protein content of over 95%. Moreover, the conjugation process had a low effect on the binding ability of R-vcMMAE to ovarian cancer cells. The results showed that the R-vcMMAE conjugate inhibited 50% of ovarian cancer cell viability at approximately 6 nM without affecting normal ovarian cell viability. In vitro studies indicated that the synthesized ADC exhibited minimal aggregation, did not adversely affect the antibody’s binding to the antigen, and displayed efficacy.

## Graphical abstract

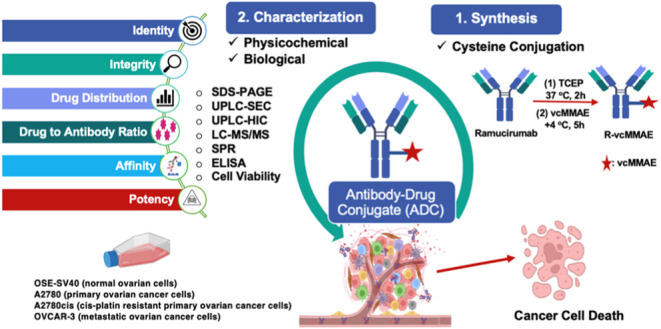

## Introduction

Ovarian cancer ranks as the seventh most prevalent cancer among women globally. As of 2020, it accounts for 314,000 new cases and 207,000 deaths annually.^1^ The high mortality rate is largely due to the challenges of early detection, leading to diagnosis at advanced stages where surgical intervention is the primary treatment. Despite treatment, recurrence and associated fatalities persist. Furthermore, the side effects of conventional chemotherapy and the emergence of drug resistance underscore the need for novel therapeutic approaches.^2–4^

Monoclonal antibodies (mAbs) are the most well-known targeted therapies. mAbs provide high specificity and affinity for the target molecule or antigen, thus enhancing efficacy while minimizing side effects. Therapeutic antibodies have been used in a wide range of diseases, including cancer, inflammatory conditions, cardiovascular diseases, and infections.^5–7^ However, mAbs are mostly used in combination with chemotherapy, limiting their therapeutic impact and highlighting the need for more potent alternatives.^8^

Antibody-drug conjugates (ADCs), which combine the potent cytotoxic effects of chemotherapy with the specificity of mAbs, represent an innovative approach to cancer treatment.^9–11^ Achieving tumor specificity in ADCs is highly dependent on the selection of the appropriate mAb. Nineteen ADC drugs have gained global approval as of 2025, primarily for the treatment of various cancers, including breast cancer, lymphomas, nonsmall cell lung cancer, bladder cancer, cervical cancer, gastric cancer, and melanoma.^12,13^ Mirvetuximab soravtansine,^14^ an ADC targeting the α-folate receptor (FRα), is the only ADC approved by the FDA for ovarian cancer.^15,16^ Ongoing clinical trials are exploring ADCs targeting FRα and sodium-dependent phosphate transport protein (NaPi2b) in ovarian cancer therapy.^15–18^

Vascular Endothelial Growth Factor (VEGF) and its receptor VEGFR-2 are key mediators of angiogenesis, making VEGFR-2 a potential target for antibody-drug conjugates (ADCs) in cancer treatment. VEGFR-2 expression has been observed in ovarian cancer cells, but not in normal ovarian cells.^19–22^ Ramucirumab (Cyramza),^23^ a fully human IgG1 monoclonal antibody that binds to the extracellular domain of VEGFR-2 with high affinity, is approved for the treatment of gastric cancer, colorectal cancer, hepatocellular carcinoma, and nonsmall cell lung cancer (NSCLC), either as a monotherapy or in combination with other drugs. While Ramucirumab is primarily recognized for its antiangiogenic activity via VEGFR-2 blockade, regulatory data indicate that it can also induce VEGFR-2 internalization in VEGFR-2–expressing cells. Specifically, a report by the Japanese Ministry of Health demonstrated that Ramucirumab reduces VEGFR-2 surface levels in PAE-KDR cells, suggesting internalization of the receptor upon antibody binding.^24,25^ Furthermore, Ramucirumab has been explored in the context of ADC development, as documented in the ADCDB database,^26^ which lists multiple Ramucirumab-based ADC candidates. These data support the feasibility of using Ramucirumab as an internalizing antibody moiety for ADC construction. We also selected Ramucirumab based on its high tumor specificity and minimal off-target toxicity, consistent with our aim of developing a selective and effective ADC.

Recent advances, as summarized in a 2025 review on ADCs in gynecologic cancers, indicate that most clinical and preclinical efforts have focused on targets such as folate receptor α (FRα), tissue factor (TF), human-epidermal receptor-2 (HER-2), trophoblast cell surface antigen 2 (Trop-2), NaPi2b, mesothelin, claudin-6 (CLDN6), cadherin-6 (CDH6), nectin-4 and B7–H4.^27^ However, to date, no ADCs targeting VEGFR-2—the molecular target of Ramucirumab—have been reported in the context of gynecologic cancers.^27,28^ This highlights a significant gap in the current ADC development landscape and underscores the rationale for investigating a novel Ramucirumab-based ADC construct for ovarian cancer therapy. Therefore, this study represents the first investigation of a Ramucirumab-based vcMMAE ADC for ovarian cancer treatment, supported by our internationally granted patent.^29^ In this study, we synthesized and characterized an antibody-drug conjugate (ADC) by conjugating Ramucirumab with the cytotoxic agent monomethyl auristatin E (MMAE) to evaluate its potential anticancer efficacy in ovarian cancer. MMAE is a synthetic antimitotic agent that inhibits tubulin polymerization, sharing a similar mechanism with taxane-based chemotherapeutics^30^ like paclitaxel, which is widely used in ovarian cancer treatment.^31^ It was also selected among several clinically validated ADC payloads—including DNA-damaging agents and maytansinoids—due to its high potency, favorable pharmacological properties^32^ and demonstrated clinical success in multiple FDA-approved ADCs.^12,13^ The conjugation was carried out using a well-characterized, lysosomally cleavable dipeptide linker composed of valine-citrulline (vc) and a self-immolative *p*-aminobenzyl carbamate (PAB) spacer. The cleavage and intracellular release mechanisms of this linker system have been extensively validated in the literature.^33,34^ The linker was attached to Ramucirumab via interchain disulfide bonds, which were formed following the partial reduction of the antibody’s native disulfide bonds by tris­(2-carboxyethyl)­phosphine (TCEP). The resulting ADC, Ramucirumab-mc-vc-PAB-MMAE (R-vcMMAE), was characterized by sodium dodecyl sulfate polyacrylamide gel electrophoresis (SDS-PAGE), ultraperformance liquid chromatography size-exclusion chromatography (UPLC-SEC), UPLC- hydrophobic interaction chromatography (UPLC-HIC), and liquid chromatography-tandem mass spectrometry (LC-MS/MS). Its binding affinity was assessed using surface plasmon resonance (SPR) and cell-based enzyme-linked immunosorbent assay (ELISA) and in vitro FcγR based ELISA.

## Experimental Section

### Synthesis of ADC

The R-vcMMAE conjugate was synthesized by targeting the interchain disulfide bonds of Ramucirumab in a 50 mM phosphate buffer containing NaCl and EDTA at pH 7.3. Initially, TCEP was added to the Ramucirumab (Cyramza, Lilly) to reduce the mAb. The reaction mixture was then incubated at 37 °C for 2 h with gentle shaking on a heater. Subsequently, excess TCEP was removed using a HiTrap desalting column packed with G25 resin (Cytiva). The column was conditioned with the reaction buffer, and elution was carried out using the same buffer, with separate collection of eluates. The concentration of the reduced mAb was determined by constructing a standard curve using reference Ramucurimab and measuring its absorbance via nanodrop. Additionally, the number of free thiol groups per mAb was determined using Ellman’s test. Following this, the reduced Ramucirumab was incubated with vcMMAE (MedChemExpress), Cat. No. (HY-15575) for 5 h under gentle shaking to facilitate the conjugation of the linker/payload to the mAb. Excess vcMMAE was removed by centrifugation (13,300 rpm for 15 min) using a 10 kDa cutoff filter (Amicon, Merck). The buffer of the synthesized ADC was exchanged to PBS, and the concentration of the ADC was determined using nanodrop. The conjugate in PBS was then stored at +4 °C.

### Sodium Dodecyl Sulfate-Polyacrylamide Gel Electrophoresis (SDS-PAGE)

Reference Ramucirumab and R-vcMMAE were diluted in ultrapure water (UPW), and the samples were combined with a loading dye. The loading dye contained DTT (BioShop) for reduced conditions. Subsequently, the samples were incubated at room temperature for nonreducing conditions and at 70 °C for reducing conditions for 10 min. The samples were then loaded and resolved on a 6–10% gradient SDS-PAGE gel. A multicolor broad-range protein marker (Thermo Spectra) was included as a reference. The gel was stained with Coomassie Brilliant blue R 250 (Merck) for 2 h. After destaining, the protein bands were visualized using the Gel DocTM XR+ System (Bio-Rad).

### Ultraperformance Liquid Chromatography Size-Exclusion Chromatography (UPLC-SEC)

The monomeric protein amounts of both reference Ramucirumab and the synthesized ADC were determined using a TSKgel UP-SW3000 SEC column (2 μm, 4.6 mm × 300 mm, Tosoh Bioscience) on a Thermo UPLC system. Analysis was performed on 10 μL samples (1 mg/mL in PBS) using a buffer containing 100 mM sodium phosphate and NaCl (pH 6.8) at room temperature. The flow rate was set to 0.35 mL/min, and detection was carried out at a wavelength of 280 nm.

### UPLC-Hydrophobic Interaction Chromatography (UPLC-HIC)

The drug-to-antibody ratio (DAR) and drug distribution were assessed using a BioPro HIC HT column (4.6 × 100 mm, 2.3 μm, YMC) on a Thermo UPLC system. Analysis of 10 μL samples (1 mg/mL in PBS) was conducted at 30 °C, employing two mobile phases: A = 20 mM sodium phosphate containing 2 M ammonium sulfate (pH 7) and B = 20 mM sodium phosphate (pH 7). Detection was performed at 280 nm.

### Liquid Chromatography-Tandem Mass Spectrometry (LC-MS/MS)

Intact mass analysis was performed using a Xevo G2-XS QTof mass spectrometer (Waters, UK) equipped with an electrospray ionization (ESI) source operated in positive ion mode. Samples were introduced via direct infusion. The acquisition range was set from 500 to 4000 *m*/*z* with a scan time of 1.0 s. The source temperature was 125 °C, capillary voltage was 3.00 kV, cone voltage was 80 V, and desolvation temperature was 350 °C with a desolvation gas flow of 800 L/h. Prior to analysis, the samples were treated with DTT. The analysis was performed utilizing an ACQUITY UPLC-BEH300 C4 column (2.1 mm × 50 mm, 1.7 μm). Two mobile phases were employed: mobile phase A consisted of water containing 0.1% formic acid (FA), while mobile phase B comprised acetonitrile (ACN) containing 0.1% FA. Data acquisition was conducted at 280 nm.

### Surface Plasmon Resonance (SPR)

Binding kinetics analysis was conducted via surface plasmon resonance (SPR) to determine the affinity constant of the antigen–antibody interaction using a Biacore T200 system (GE Healthcare). Initially, immobilization of the VEGFR-2 ligand (R&D Systems, Cat. No. 357-KD-050/CF) on a CM5 chip (GE Healthcare, Cat. No. BR-1005–30) surface was achieved through amine coupling. The chip surface was activated with 1-ethyl-3-(3-(dimethylamino)­propyl) carbodiimide (EDC) and sulfo-NHS using the CYTIVA amine coupling kit. Subsequently, both the reference Ramucirumab and the synthesized R-vcMMAE were injected onto the ligand-coated surfaces in HBS-EP buffer (GE Healthcare, Cat. No. BR-1006–69) at nanomolar concentrations. The binding affinity constant (KD, M) was calculated from the global binding kinetics using a kinetic binding model in the evaluation software of the Biacore T200 instrument, fitted to the 1:1 Langmuir binding model. KD measurements were performed in two independent experiments (*n* = 2).

### Cell Culture: The Ovarian Cells were Obtained from the European Collection of Authenticated Cell Cultures (ECACC)

Normal ovarian surface epithelial cell line (OSE-SV40) and three ovarian cancer cell lines (A2780, A2780cis, and OVCAR–3) were cultured in RPMI (Sigma) supplemented with 10% FBS (Gibco) and 1% penicillin-streptomycin (P/S, Gibco) at 37 °C with 5% CO2. Upon reaching 70–90% confluence, the cells were trypsinized and counted using trypan blue.

### Cell Based Enzyme-Linked Immunosorbent Assay (ELISA)

To evaluate the binding effect of the linker/drug conjugation to the monoclonal antibody (mAb), cell-based ELISA was employed. 50,000 cells/well were seeded in clear 96-well plates at 100 μL per well. After overnight incubation, the culture media was aspirated, and a fixing solution (4% formaldehyde solution in PBS) was added at 100 μL per well to fix the cells. Following a 30 min incubation at room temperature, wash buffer (0.1% Tween-20 in PBS) was used for three washes, each with 200 μL per well, to remove the fixing solution. Blocking buffer (2% BSA, 0.5% Triton X-100 in PBS) was then added at 200 μL per well and incubated for 1 h at room temperature. Subsequently, the wells were washed three times to remove the blocking buffer. Reference Ramucirumab and R-vcMMAE at concentrations ranging from 2 to 0.03 μM were added at 50 μL per well. After overnight incubation at 4 °C, the wells were washed three times. Next, an HRP-conjugated secondary antibody (Life Technologies) was added at 50 μL per well and incubated for 90 min in the dark. Following three washes with wash buffer, 50 μL of TMB substrate (Thermo Scientific) was added to each well. After approximately 20 min, when a blue color developed, 50 μL of stop solution (0.18 M H_2_SO_4_) was added to halt color development. The optical density was measured at 450 nm.

### Cytotoxicty Assay

To investigate the effect of the synthesized ADC, a cell viability assay was conducted by comparing the potency of R-vcMMAE with reference Ramucirumab, MMAE, and paclitaxel. 10,000 cells/well were seeded in clear 96-well plates at 100 μL per well and allowed to grow overnight. Reference Ramucimab, R-vcMMAE, MMAE, and paclitaxel at various concentrations were added at 50 μL per well into the growth media, respectively. Control wells did not receive any drug treatment. After 72 h of exposure to treatment, the cells in the wells were allowed to equilibrate to room temperature, and the media was carefully aspirated. RPMI medium without supplements was then added to each well at 50 μL. Subsequently, CellTiter-Glo (Promega) reagent was added at a 1:1 (v/v) ratio over the media. The cells were shaken at 250 rpm for 30 min on an orbital shaker in the dark. Luminescence emitted by the lysed cells was measured in a black 96-well plate using a microplate luminometer (Berthold Centro XS3 LB 960).

Percent viability was calculated according to the following equation

(average sample wells/average control wells)×100

### In Vitro FcγR Based ELISA

Recombinant human Fcγ Receptors (FcγRs) containing FcγRIIa H131 (SinoBiological) and FcγRIIIa V158 (SinoBiological) were diluted in coating buffer, which consisted of 70% 0.1 M sodium bicarbonate and 30% 0.1 M sodium carbonate. The receptors were then coated onto a 96-well ELISA plate at a concentration of 0.5 μg/mL and incubated at 4 °C overnight. Following this, blocking buffer (2% BSA, 0.5% Triton X-100 in PBS) was added to each well and incubated for 2 h at room temperature. Reference Ramucirumab and R-vcMMAE at concentrations ranging from 0.551 to 0.0008 μM were subsequently added and incubated for 2 h. Later, an HRP-conjugated secondary antibody (Life Technologies, Cat. No. H10307) was added and incubated for 90 min in the dark. Washing steps were performed between each step using wash buffer (0.05% Tween-20 in PBS). Subsequently, TMB substrate (Thermo Scientific) was added, and color development was allowed to occur. To stop color development, stop solution (0.18 M H_2_SO_4_) was added to the TMB solution. The optical density was then measured at 450 nm.

### Statistical Analysis

Data are presented as the mean ± standard deviation (SD) of at least three independent experiments, with each experiment performed in triplicate. Statistical analyses and graph generation were conducted using GraphPad Prism version 10 (GraphPad Software, San Diego, CA). Comparisons between two groups at corresponding concentrations were evaluated using unpaired two-tailed Student’s *t* tests. Statistical significance was represented as follows: ns (not significant), * (*p* < 0.05), ** (*p* < 0.01), *** (*p* < 0,001), or **** (*p* < 0,0001) in the figure.

## Results and Discussion

The selection of mAb, payload, and linker is crucial for optimizing the therapeutic potential of ADCs. Angiogenesis-targeted ADCs are emerging as a promising strategy for advancing therapeutics to treat ovarian cancer patients. Ramucirumab, an IgG1 antibody targeting VEGFR-2, possesses four interchain disulfide bonds, two between heavy chains and two between heavy and light chains, located in the flexible hinge region. These bonds can be partially reduced without unfolding the antibody structure, facilitating cysteine conjugation and allowing for controlled Drug Antibody Ratio (DAR) without necessitating extensive antibody modification.^35^ Ramucirumab effectively targeted ovarian cancer cells, as VEGFR-2 expression was detected in approximately 75% of invasive ovarian cancer samples but was minimal or absent in normal ovarian epithelial cells,^19^ highlighting its suitability for ovarian cancer therapy. Furthermore, previous work from our laboratory^36^ reported that VEGFR2 mRNA expression was elevated in ovarian cancer cell lines (A2780, A2780cis, and OVCAR-3) compared to healthy epithelial OSE-SV40 cells.

In our study, mc-val-cit-pab-MMAE was conjugated to Ramucirumab following partial reduction of interchain disulfide bonds on the mAb. R-vcMMAE, like Mirvetuximab soravtansine, utilizes conventional conjugation methods, which are quicker, cost-effective, and easier to scale compared to more complex site-specific conjugation techniques. The high therapeutic index, low aggregation, and potent anticancer activity of R-vcMMAE further validate the efficacy of this approach for ovarian cancer treatment. Although Mirvetuximab soravtansine has shown clinical promise, R-vcMMAE offers a different mechanism by targeting VEGFR-2, suggesting that it may complement existing therapies or offer an alternative strategy for ovarian cancer patients who do not respond well to other treatments. The toxicity of the ADC arises from its payloads. MMAE, at nanomolar concentrations, can readily penetrate cells through passive diffusion as a free, nonspecific entity.^37^ Additionally, the valine-citrulline linker is designed to cleave in the presence of lysosomal protease cathepsin B, ensuring payload protection until reaching target cancer cells and providing stability in circulation.^38^ Moreover, the linker design ensured that MMAE was selectively delivered to the cancer cells, protecting healthy tissues from the toxic effects of the payload. The linker’s stability in circulation, along with its cleaving mechanism in the lysosome, facilitated controlled drug release in the target cells, contributing to the safety profile of the ADC.^10,39,40^ Therefore, the chosen linker in this study appears well-suited for conjugating the highly toxic antimitotic drug, MMAE, to Ramucirumab. Given MMAE’s high potency and rapid clearance rate, the payload has been loaded at a lower level, as determined by spectrometric and chromatographic methods. The synthesized ADC, R-vcMMAE, was characterized by Ellman test, SDS-PAGE, UPLC-SEC, UPLC-HIC, and LC-MS/MS. The binding affinity of R-vcMMAE was assessed using SPR and ELISA. Furthermore, the anticancer activity of R-vcMMAE was evaluated on normal ovarian cell line (OSE-SV40) and ovarian cancer cell lines (A2780, A2780cis, and OVCAR-3) using cell viability assays to assess its potential as a therapeutic agent for ovarian cancer. The schematic representation of the synthesized R-vcMMAE is shown in Figure 1.

**Figure 1:**
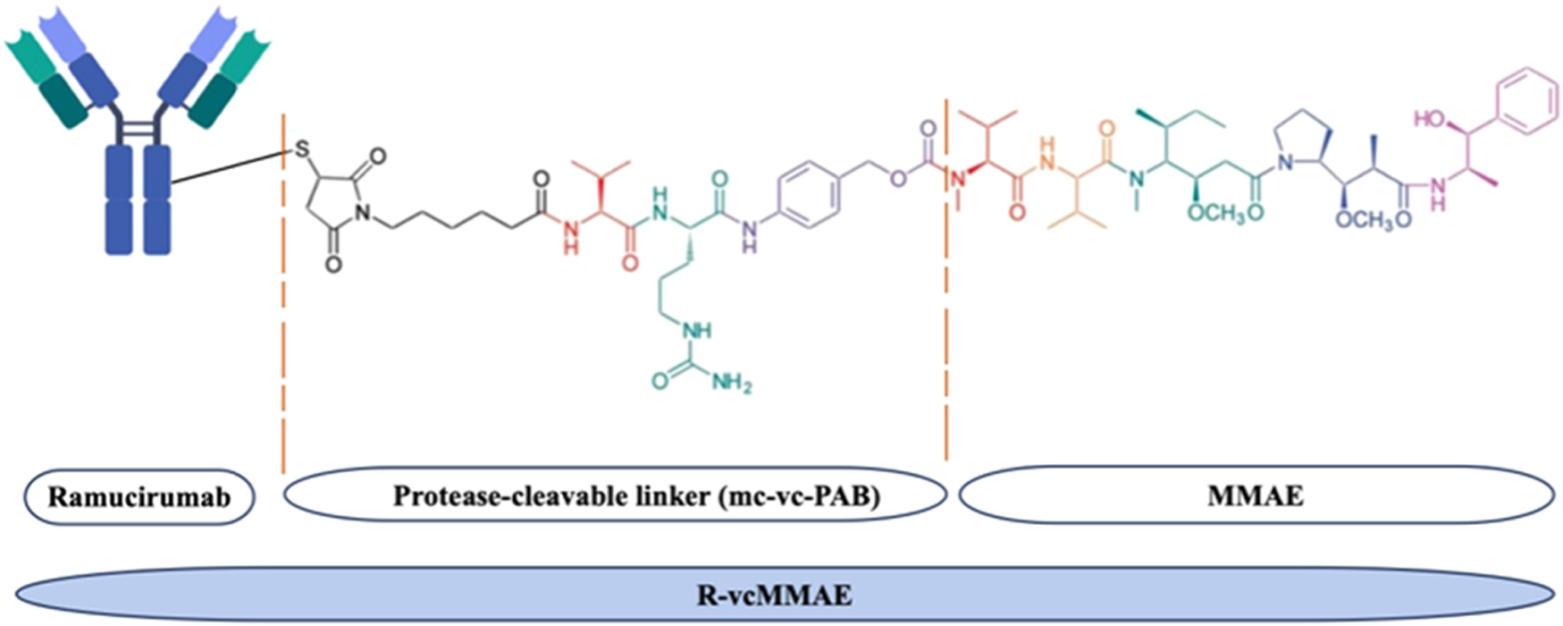
Schematic demonstration of R-vcMMAE [modified from ref 41]. The antimitotic agent MMAE was conjugated to Ramucirumab via a lysosomally cleavable dipeptide linker, containing valine-citrulline and a self-immolative *p*-aminobenzoyloxycarbonyl (PAB) group.

Ramucirumab was first subjected to partial reduction of disulfide bonds using TCEP to enable conjugation. Once the reduction process was complete, an internal control analysis was performed using the Ellman assay. A standard curve was generated using known concentrations of cysteine by measuring absorbance at 412 nm. This curve, which is shown as an inset in Figure 2, demonstrates a strong linear correlation (*R* ^2^ = 0.99), indicating that cysteine concentration can be accurately quantified. The amount of free thiol groups quantified by the Ellman assay allows for the estimation of the DAR of the synthesized ADC. Free thiol groups per antibody in the sample were measured using 5,5′-dithiobis­(2-nitrobenzoic acid) (DTNB), which reacts with sulfhydryl groups to produce a yellow-colored product, 2-nitro-5-thiobenzoic acid (TNB), detectable at 412 nm. The reaction buffer containing Ellman’s reagent without the sample was used as a blank. An extinction coefficient of ε412 = 1.4 × 10^5^ M^–1^cm^–1^ was applied to calculate thiol concentrations.^37^ As shown in Figure 2, disulfide bonds of Ramucirumab were partially reduced and then alkylated with vcMMAE demonstrating successful ADC synthesis. Furthermore, the mean number of free thiols was calculated as 4.1.

**Figure 2:**
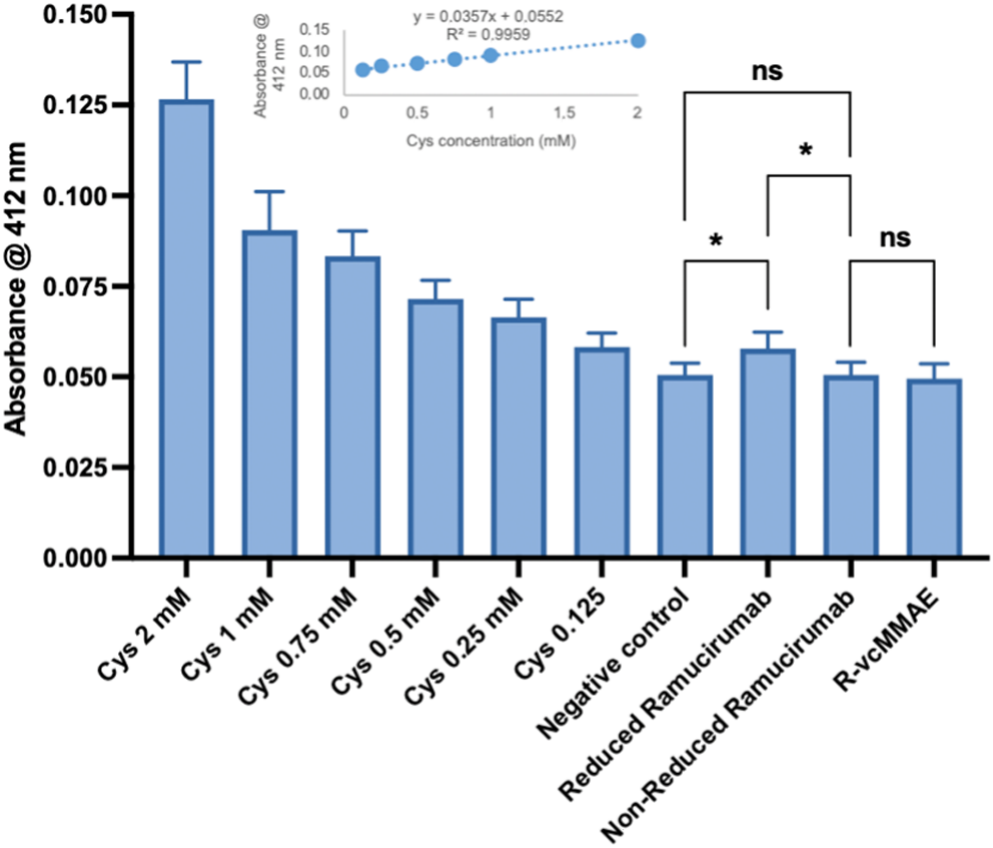
Ellman’s test. The test was performed to determine both free thiols per mAb and the alkylation confirmation at 412 nm. Cysteine (Cys) as positive control and reaction buffer as negative control were used. Reduced Ramucirumab refers to the TCEP-treated antibody prior to conjugation, while “R-vcMMAE” refers to the antibody after conjugation. (Error bar showed SD of at least three independent experiments. Student’s *t* test was used to determine the significant difference).

The analysis was carried out under reducing and nonreduced conditions for Ramucirumab and R-vcMMAE (Figure 3). Both ADC (Lane 1) and reference mAb (Lane 2) gave two bands under reduced conditions at 50 kDa for heavy chain and 25 kDa for light chain, respectively, showing their integrity. A protein marker ranging from 260 kDa to 10 kDa was loaded into Lane 3. While reference mAb gave a band at 150 kDa (Lane 4) under nonreduced conditions, the ADC had six bands (Lane 5) at nearly 150, 125, 100, 75, 50, and 25 kDa corresponding to two heavy-two light chain (H2L2), two heavy-one light chain (H2L), two heavy chain (H2), one heavy-one light chain (HL), heavy chain (H), and light chain (L), respectively; representing drug distribution due to utilizing cysteine conventional conjugation method. The degree of observable H2L2 form was notably low compared to others, likely attributed to the alkylation of some interchain disulfide bonds by vcMMAE.^42^ The additional bands observed under nonreducing SDS-PAGE likely correspond to partially conjugated or aggregated antibody species, which is typical in nonsite-specific thiol-based conjugations.^9,41^ However, our SEC-HPLC analysis demonstrated a monomeric purity of over 95%, indicating minimal aggregation and that the ADC preparation is largely free from aggregates (Figure 4). These findings for SDS-PAGE analysis regarding drug distribution on the reference mAb align with similar studies that utilized conventional methods targeting cysteine residues of the mAb.^9,43,44^

**Figure 3:**
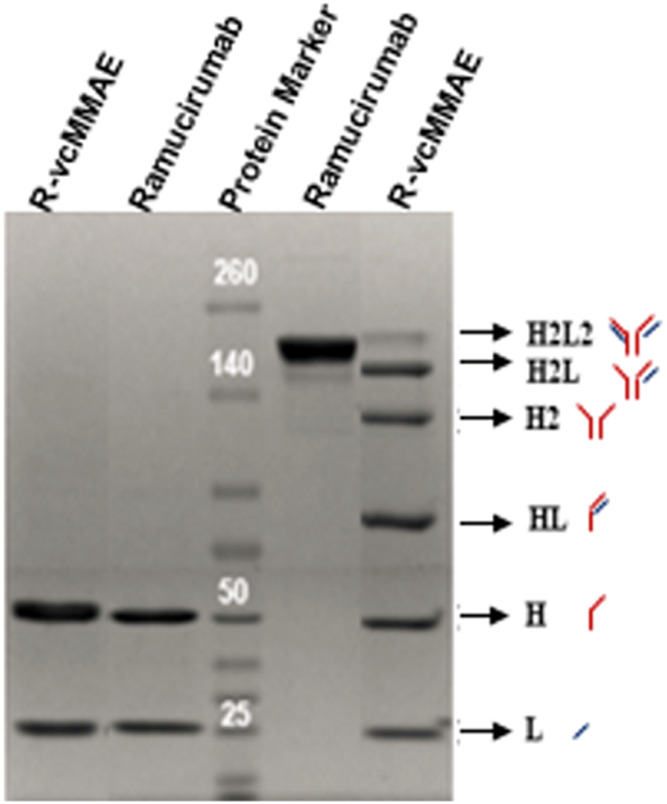
SDS-PAGE analysis of Ramucirumab and R-vcMMAE under reducing (left of protein marker) and nonreducing (right of protein marker) conditions. The samples were resolved on a 6–10% gradient SDS-PAGE gel.

**Figure 4:**
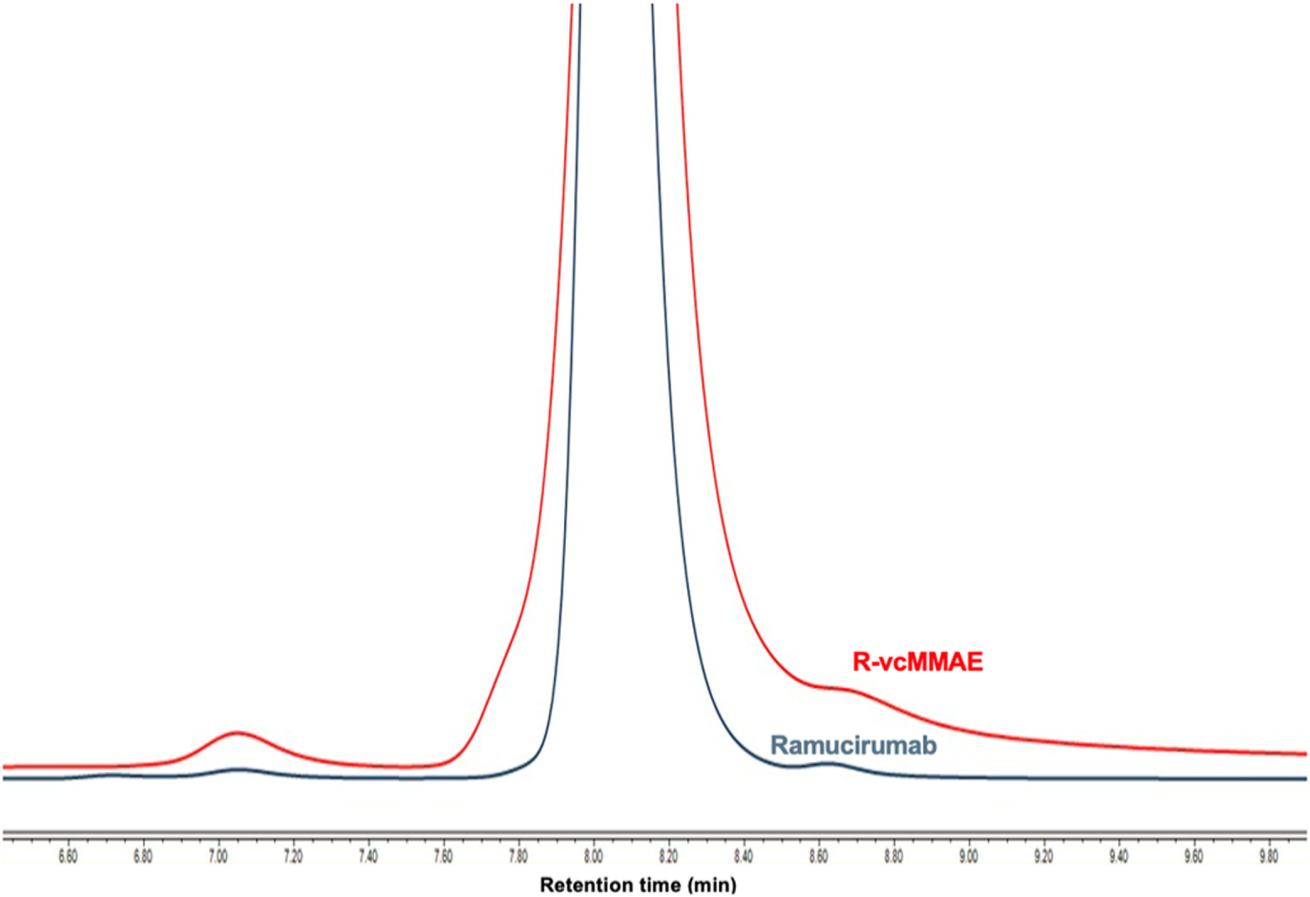
UPLC-SEC analysis of Ramucirumab (rt: 8046 s) and R-vcMMAE (rt: 8079). Column: TSKgel UP-SW3000 SEC column (2 μm, 4.6 mm × 300 mm, Tosoh Bioscience) Mobil phase: 100 mM sodium phosphate and NaCl (pH 6.8).

The intact construct of synthesized R-vcMMAE was also confirmed by UPLC-SEC (Figure 4). The original full chromatogram was provided as Figure S1. The chromatograms of reference mAb and synthesized ADC demonstrated that slight retention time (rt) changes and peak tailing. However, there was no peak at longer elution times, showing no significant aggregation. Besides, the monomeric peak of synthesized R-vcMMAE is higher than 95%. The tailing can be due to interaction between the payload and the stationary phase of the column. These results suggested that the conjugation process did not affect the integrity and there was no dissociation of the chain.

R-vcMMAE was characterized by UPLC-HIC to assess the information about drug distribution and calculate the DAR value. Figure 5 showed that the synthesized ADC contained a mixture of species ranging from 0 to 6 drug conjugation. The mean DAR value was calculated from the integration of each peak and determined as 3.2. The species of DAR = 4 presented the majority having more than 45% (Table 1).

**Figure 5:**
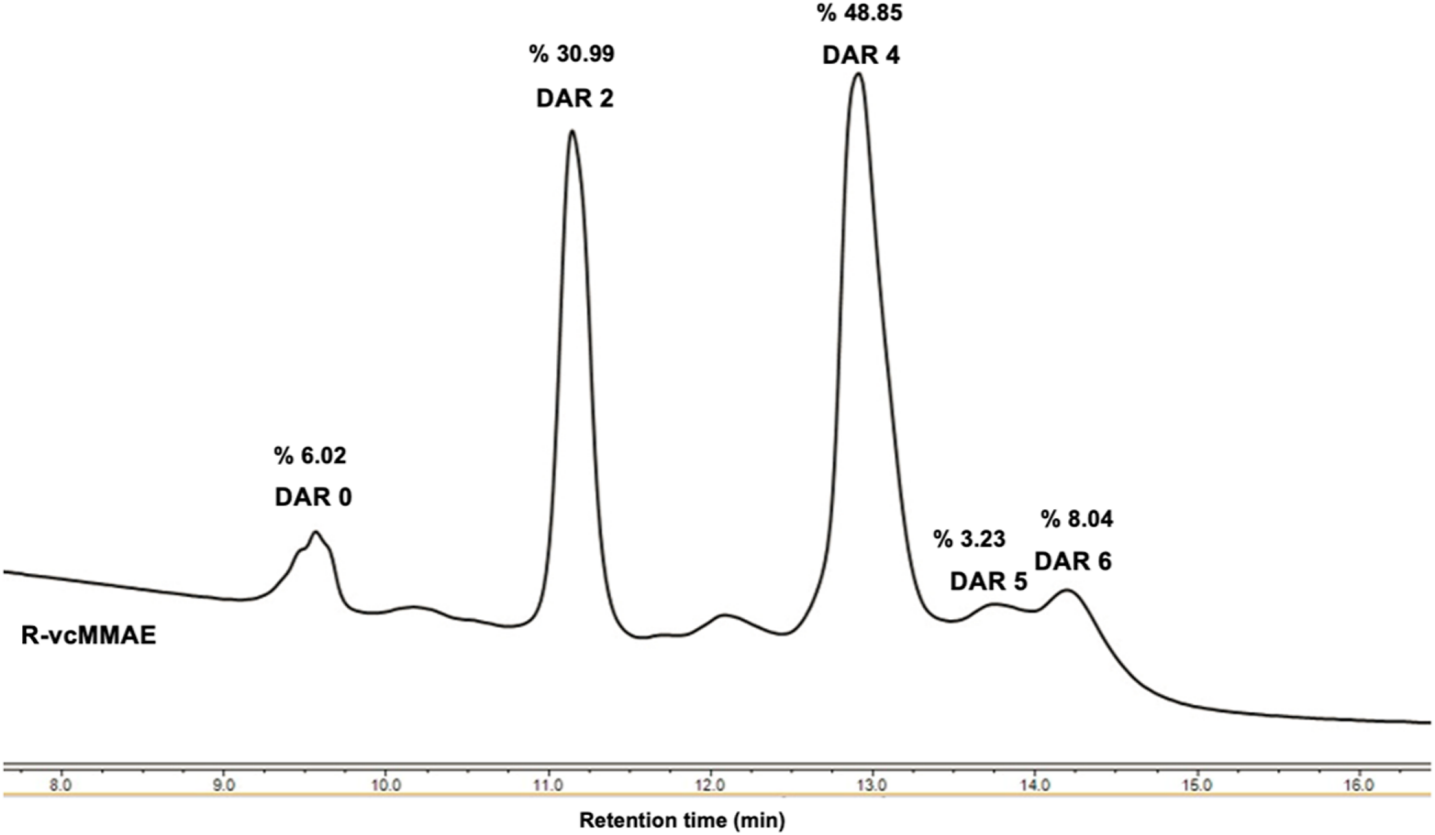
UPLC-HIC analysis of R-vcMMAE. Column: BioPro HIC HT column (4.6 × 100 mm, 2.3 μm, YMC) Mobile phase A = 20 mM sodium phosphate containing 2 M ammonium sulfate (pH 7) and Mobile phase B = 20 mM sodium phosphate (pH 7).

**Table 1:** DAR Determination Based on Relative Peak Area of Each Loaded Species According to HIC Data

DAR											
	0		2		4		5		6		
	area (%)	DAR contribution	area (%)	DAR contribution	area (%)	DAR contribution	area (%)	DAR contribution	area (%)	DAR contribution	total DAR
R-vcMMAE	6.02	0	30.99	0.62	48.85	1.95	3.23	0.16	8.04	0.48	3.2

The cysteine conjugation method employed in ADC studies theoretically allows for zero to eight drugs per mAb,^43,44^ with the DAR being contingent on the conjugation process. Consequently, the synthesized ADC exhibited over 94% monomeric peak and had an approximate DAR value of 3.2. Furthermore, published studies suggest that ADCs with a DAR value of 4, synthesized over cysteine residues, yield the most favorable therapeutic effects,^41,45,46^ which is consistent with our findings, demonstrating more than 45% of species with a DAR = 4.

Each peak in HIC spectra confirmed that each species has a different number of drug conjugates, appearing as an intact and the unconjugated Ramucirumab after the conjugation process was about 6% in a mixture.

Mass spectrometry was employed to determine the specific chain to which the drug was bound and the number of conjugated molecules. Mass spectrometric analysis of reduced ramucirumab (Figure 6A) showed major peaks corresponding to the light chain (L, ∼23.3 kDa) and heavy chain (H, ∼46.6 kDa). In the conjugated R-vcMMAE sample (Figure 6B), the light chain displayed a mass increase of ∼1.46 kDa (L + 1), consistent with the addition of a single mc-vc-PAB-MMAE payload (theoretical mass ∼ 1.32 kDa). The heavy-chain region displayed multiple masses, including a major peak at ∼ 53.3 kDa (+∼6.7 kDa from H), consistent with higher drug load states and additional peaks attributable to partially conjugated species. Despite these shifts, the calculated average drug-to-antibody ratio (DAR) was approximately 3–4, suggesting heterogeneity in conjugation, consistent with results from HIC (Figure 5). SDS-PAGE also supported conjugation heterogeneity, showing multiple bands characteristic of diverse drug loading states (Figure 3). This discrepancy between observed drug load and calculated DAR may be attributed to differential ionization efficiency or partial loss of payload. In the deconvoluted MS spectra, minor peaks showing +162 and +203 Da shifts were observed, suggesting the presence of natural glycoform variants, as glycan structures remain unaltered in this conjugation approach. Additionally, +22 and +38 Da adducts corresponding to sodium and potassium ions were detected, likely due to sample buffer or LC-MS solvent contamination, which commonly causes such adduct formation. These complementary findings confirm successful conjugation and illustrate the inherent heterogeneity of cysteine-based ADCs.

**Figure 6:**
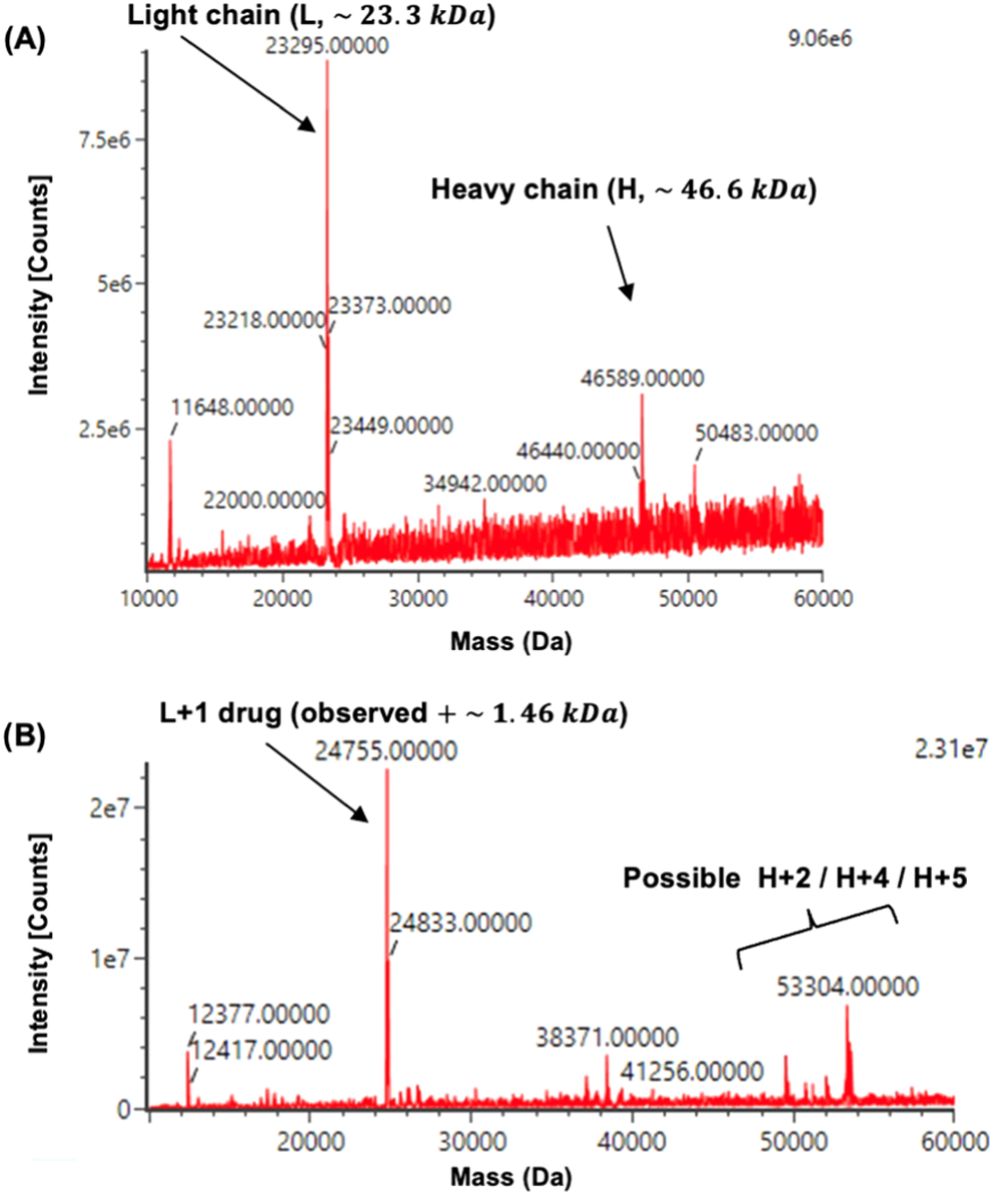
Deconvolved MS spectra for (A) Ramucirumab and (B) synthesized R-vcMMAE. Column: ACQUITY UPLC-BEH300 C4 column (2.1 mm × 50 mm, 1.7 μm). Mobile phase A = Water containing 0.1% FA, Mobile phase B = ACN containing 0.1% FA.

The DAR of R-vcMMAE was found to be approximately 3.2, which is consistent with the typical DAR values observed in other clinically approved ADCs, such as Mirvetuximab soravtansine, which has a DAR of approximately 3.5.^47^ Studies have shown that a DAR between 2 and 4 is optimal for achieving therapeutic efficacy without significant aggregation or off-target effects.^43–45^ The lower DAR in R-vcMMAE still showed potent anticancer activity, which aligns with the notion that ADCs with a lower DAR may still exhibit strong therapeutic effects without compromising safety and specificity.

After verifying and determining structural and physicochemical properties of synthesized R-vcMMAE, binding affinity toward soluble VEGFR-2 antigen was evaluated by SPR. In the analysis, the equilibrium dissociation constant (KD) is related to the ligand affinity to the receptor and the lower KD indicates a higher affinity. The KD value less than nanomolar indicates a strong binding affinity between an antibody and its antigen. This high binding affinity signifies effective localization of the antibodies on tumor cells, offering target specificity.^10,48,49^ As depicted in Table 2, the KD value of the synthesized ADC was 10.8 ± 1.4 pM, which was comparable to that of the reference Ramucirumab (12.2 ± 2.3 pM), based on two independent experiments. The result presented that the drug conjugation to the mAb process did not influence the affinity of R-vcMMAE to the target antigen, exhibiting high binding affinity to the target antigen. The corresponding SPR sensorgrams and binding curves used for KD determination have been provided in Figure S2.

**Table 2:** SPR Analysis Results for Reference Ramucirumab and R-vcMMAE

	KD^a^ (pM)	
Ramucirumab	10.58	13.85
R-vcMMAE	9.82	11.86

a KD = Binding Affinity Constant

The cell-based ELISA assay was used to investigate whether drug conjugation affects the VEGFR-2 binding affinity of the reference mAb on the surface of ovarian cancer cells. The affinity depended on the concentration in a manner (Figure S3). There was a negligibly binding affinity reduction on the metastatic cancer cell line for R-vcMMAE when compared to the reference mAb. R-vcMMAE is a promising ADC for ovarian cancer therapy that combines VEGFR-2 targeting with the cytotoxic payload MMAE. Its strong anticancer activity, coupled with minimal off-target effects, positions it as a potential candidate for further clinical development alongside other FDA-approved ADCs such as Mirvetuximab soravtansine and Bevacizumab.

The cell viability assay was performed to assess the antitumor activity of R-vcMMAE on normal ovarian cell OSE-SV40, and ovarian cancer cell lines A2780, A2780cis and OVCAR-3. The conjugate was compared with unconjugated mAb, cytotoxic drug in conjugate MMAE and commercial drug paclitaxel. The results demonstrated MMAE conjugation caused significant cancer cell death in nM concentration without affecting normal ovarian cells after 72 h exposure, as shown in Figure 7. Moreover, unconjugated mAb revealed negligible or no antitumor activity on both normal and cancer ovarian cells while MMAE and paclitaxel gave a nonspecific inhibitory effect. Besides, paclitaxel was effective above 40 nM on both normal and primary ovarian cells, but it did not show a significant inhibitory effect on the metastatic ovarian cancer cell line (OVCAR-3) until 1000 nM. Nevertheless, the R-vcMMAE conjugate had a half maximal inhibitory concentration (IC50) value at approximately 6 nM for primary, cis-platin-resistant primary and metastatic ovarian cancer cells without indicating any toxicity on normal ovarian cells. The approximate IC_50_ values (nM) of the drugs in four ovarian cell lines are presented in Table S1. The cell viability of OSE-SV40 started to decrease above 6.4 nM in a concentration dependent manner but target-specific toxicity was protected. The results of the luminescent cell viability assay, which quantifies ATP as a marker of metabolically active cells, demonstrated the specific killing of ovarian cancer cells by our novel ADC construct. To further evaluate the specificity of the ADC’s cytotoxicity, an additional control experiment was performed in which cells were treated with a combination of unconjugated ramucirumab and free MMAE. As expected, this combination did not demonstrate targeted cytotoxicity, supporting that the observed effects in the ADC group result from the targeted delivery mechanism. The results of this experiment are provided in Figure S4. The observed potency in ovarian cancer cells was attributed to the targeted delivery of MMAE, underscoring the selectivity achieved by the ADC.

**Figure 7:**
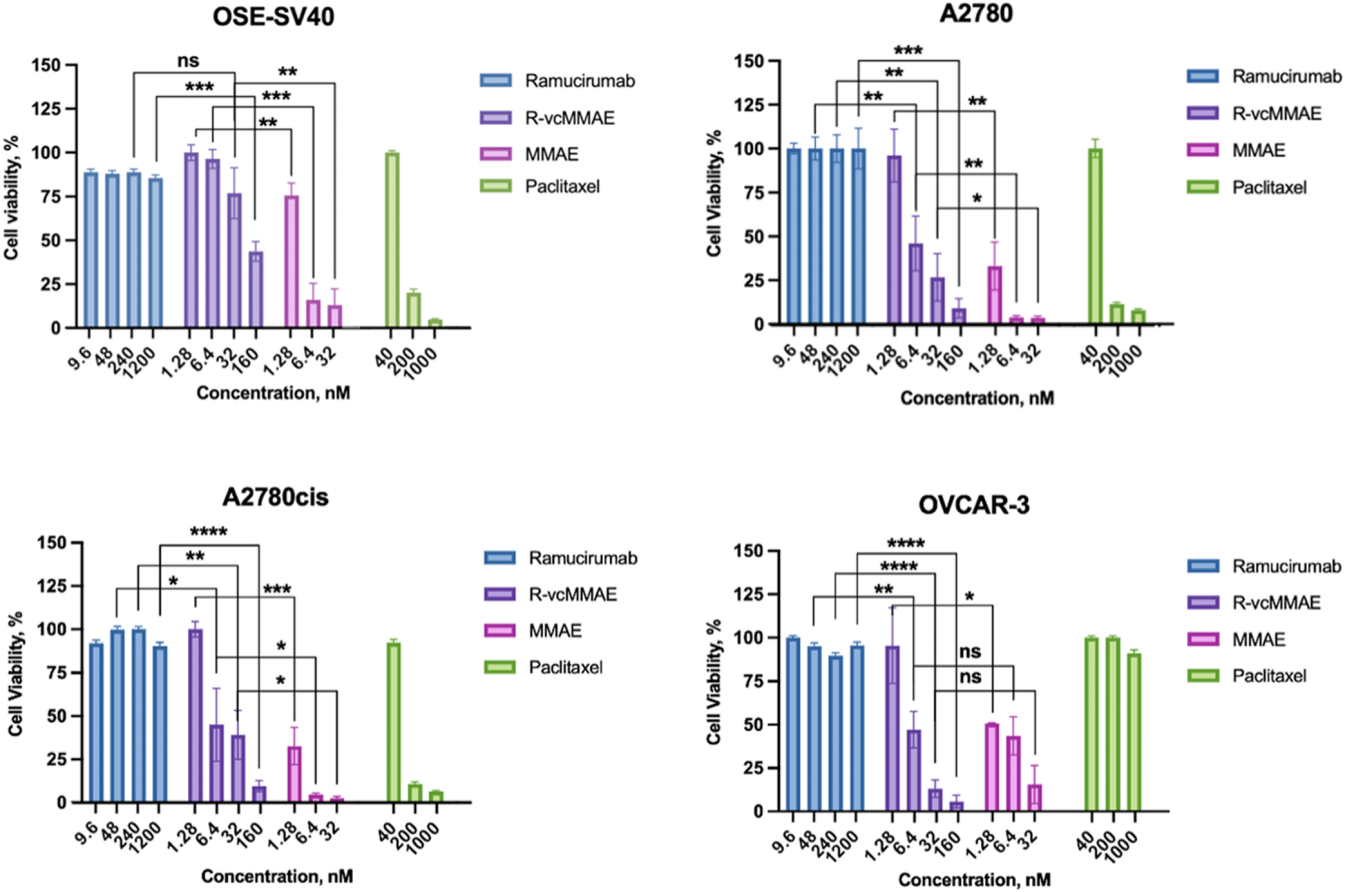
Cell viability analysis of Ramucirumab, R-vcMMAE, MMAE and paclitaxel on normal cell line (OSE-SV40), primary (A2780), cis-platin resistant primary (A2780cis) and metastatic (OVCAR-3) ovarian cell lines. (Error bar showed SD of at least three independent experiments. Student’s *t* test was used to determine the significant difference).

Ovarian cancer treatment has seen significant advancements with the approval of only ADC Mirvetuximab soravtansine, which delivers the cytotoxic agent cytotoxic maytansinoid effector molecule DM4 selectively to folate receptor α (FRα)-positive cancer cells. The ability of Mirvetuximab soravtansine to target FRα-positive cells highlights a significant therapeutic strategy in ovarian cancer, although the response to this ADC can vary based on the expression levels of FRα and the stage of the disease.^15–18,47^ In contrast, our study explored Ramucirumab-vc-MMAE (R-vcMMAE), an ADC targeting VEGFR-2. While Mirvetuximab soravtansine focuses on targeting FRα, R-vcMMAE exploits angiogenesis inhibition through the targeting of VEGFR-2, which plays a critical role in tumor vascularization and growth. The anticancer activity of R-vcMMAE against A2780, A2780cis, and OVCAR-3 suggests that it could provide a valuable therapeutic approach, especially for tumors that may not express high levels of FRα but still rely on VEGFR-2-mediated angiogenesis for growth.

Compared to Mirvetuximab soravtansine and Bevacizumab, R-vcMMAE demonstrated significant anticancer activity at nanomolar concentrations while sparing normal ovarian cells from toxicity. This is an important distinction, as Mirvetuximab soravtansine also targets cancer cells but primarily relies on FRα expression, and its activity is highly dependent on the level of FRα expression on tumor cells.^47^ Our findings with R-vcMMAE suggest that targeting VEGFR-2 may provide an alternative pathway for effective ovarian cancer therapy, especially for patients.

In the context of cell potency tests, an analysis was conducted to determine the Fcγ receptor binding capacities of antibodies. FcγRs play a crucial role in orchestrating a balanced immune response by either activating (FcγRI, FcγRIIa, and FcγRIIIa) or inhibiting (FcγRIIb) cellular responses upon binding of antibodies. Activation of FcγRs initiates intracellular signaling pathways following the cross-linking of extracellular ligand-binding domains by IgG immune complexes. Notably, FcγRIIa and FcγRIIIa receptors are known to be overexpressed in various innate immune cells, including monocytes and macrophages.^50^ Determining the FcγRIIa and FcγRIIIa binding affinities of antibodies and antibody-drug conjugates (ADCs) is crucial for evaluating Antibody-Dependent Cell-Mediated Cytotoxicity (ADCC) activity.

In vitro ADCC and ADCP activities of the synthesized ADC were assessed in comparison to Ramucirumab using an ELISA-based FcγR binding analysis. The binding affinity of Ramucirumab and R-vcMMAE toward human FcγR variants containing FcγRIIa H131 and FcγRIIIa V158 was analyzed. It was known that the FcγRIIa H131 variant is a high responder for IgG1 antibody increasing ADCP function; on the other hand, the FcγRIIIa V158 is a medium responder for IgG1 antibody, showing moderate ADCC activity.^48^ The synthesized R-vcMMAE had a significant increment in the ADCC and ADCP activity above 60 nM; however, it retained a comparable activity with unconjugated Ramucirumab below 60 nM, which includes the IC50 value of the ADC concentration (Figure S5). This means that the ADC did not produce an adverse effect on in vitro antibody-dependent activity correlated with off-target cytotoxicity. The results indicated that the ADC exhibits a concentration-dependent high affinity for both FcγRIIa and FcγRIIIa variants, surpassing the binding affinity of Ramucirumab. Notably, an increase in ADCC and Antibody-Dependent Cellular Phagocytosis (ADCP) activities was observed after reaching a concentration of 60 nM, potentially associated with the initiation of ADC aggregation at higher concentrations. It is known that aggregation of antibodies can trigger ADCC and ADCP, potentially leading to off-target cytotoxicity.^51^

The binding affinity of R-vcMMAE to VEGFR-2 was maintained postconjugation, which is consistent with findings from Bevacizumab (Avastin), another FDA-approved VEGF/VEGFR targeting agent. Bevacizumab is approved for the treatment of various cancers, including ovarian cancer, and works by inhibiting angiogenesis to block the formation of new blood vessels required for tumor growth.^52,53^ While Bevacizumab is not an ADC, its mechanism of action is similar in that it targets the VEGF/VEGFR pathway, which is crucial for tumor survival and metastasis. The potent anticancer effects observed with R-vcMMAE suggest that it may offer an enhanced approach, combining VEGFR-2 targeting with the cytotoxic effects of MMAE.

## Conclusions

The ADC was synthesized using conventional conjugation methods, which are known for their speed, ease, and cost-effectiveness compared to site-specific conjugation techniques. In this study, vc-MMAE was successfully conjugated to Ramucirumab. Structural and physicochemical characterization confirmed the successful synthesis of R-vcMMAE, with an approximate DAR value of 3.2 and less than 5% aggregation. Biochemical characterization revealed that payload conjugation did not significantly affect the binding affinity of the antibody to the antigen. Additionally, the synthesized ADC demonstrated potent activity against ovarian cancer cells at a nanomolar concentration while sparing healthy ovarian cells from harm. Furthermore, the applied IC50 dose (approximately 6.8 nM) of the ADC exhibited lower FcγRIIa H131 and FcγRIIIa V158 binding capacity, indicating preserved target-specific activity without aggregation.

In conclusion, our research introduces a novel antibody drug conjugate designed to target ovarian cancer cells and a comprehensive in vitro characterization of the synthesized ADC. The results suggest that R-vcMMAE is a promising candidate for ovarian cancer patients based on in vitro experiments. However, further investigations through in vivo experiments are necessary to validate these findings. Moreover, the newly synthesized ADC may hold potential applications in the treatment of other malignancies characterized by VEGFR-2 overexpression.

## Limitation of the Study

The primary aim of this study was to synthesize and characterize a novel ADC targeting a receptor that has not been widely explored in ovarian cancer but holds promise as a potential therapeutic target. Initial in vitro assays, including cytotoxicity evaluations, were conducted to demonstrate the functionality of the synthesized ADC. However, these preliminary in vitro studies should be further expanded to include additional functional assays such as internalization studies, lysosomal trafficking, and apoptosis analyses to gain deeper insight into the ADC’s mechanism of action.

Although the in vitro cell viability results presented in Figure 7 provide indirect evidence of intracellular MMAE release, as indicated by the selective cytotoxicity of the ADC in cancer cells compared to the nonspecific toxicity of free MMAE and the minimal effect of the unconjugated antibody, no direct experimental data confirming lysosomal cleavage or intracellular release of MMAE were included in this study. Therefore, further mechanistic studies are needed to validate this aspect.

While the synthesis and characterization of R-vcMMAE present a promising therapeutic approach, comprehensive preclinical evaluations are required to fully explore its clinical potential.

## Supplementary Material



## Notes

(Figure S1) UPLC-SEC analysis of Ramucirumab and R-vcMMAE; (Figure S2) surface plasmon resonance (SPR) sensorgrams of the reference antibody and the synthesized R-vcMMAE; (Figure S3) cell-based ELISA results for Ramucirumab and R-vcMMAE on primary (A2780), cis-platin resistant primary (A2780cis) and metastatic (OVCAR-3) ovarian cancer cell lines; (Figure S4) cell viability analysis following treatment with a combination of unconjugated ramucirumab and free MMAE; (Figure S5) FcγRs binding profile of Ramucirumab and R-vcMMAE Table S1 IC_50_ values (nM) of ramucirumab, R-vcMMAE, MMAE, and paclitaxel in ovarian cell lines (PDF)
