# Supplementary material for: Characterization of Synthesized Ramucirumab-vcMMAE as a Potential Therapeutic Approach in Ovarian Cancer
Source: ACS Omega. 2025 Aug 27;10(35):39912–22. doi: 10.1021/acsomega.5c03733 (PMC12423901; doi:10.1021/acsomega.5c03733)
Supplement: Supplementary file 1 [file ao5c03733_si_001.pdf]

Characterization of Synthesized Ramucirumab-vcMMAE as a Potential Therapeutic Approach in Ovarian Cancer

Duygu Erdogan [1,2], Hulya Ayar Kayali [1,2,3] *

[1] Izmir International Biomedicine and Genome Institute, Dokuz Eylül University, Izmir, Türkiye

[2] Izmir Biomedicine and Genome Center, Izmir, Türkiye

[3] Department of Chemistry, Division of Biochemistry, Faculty of Science, Dokuz Eylül University, Izmir, Türkiye

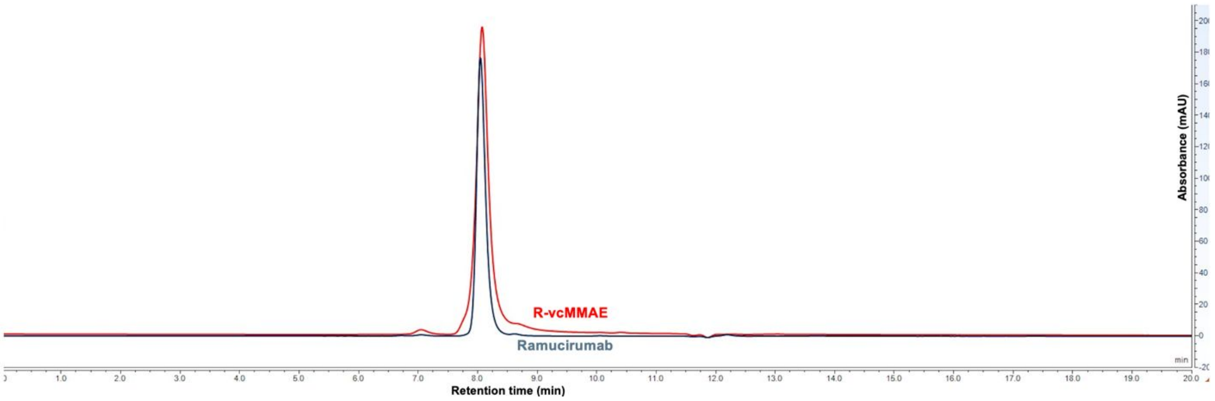

Figure S1 UPLC-SEC analysis of Ramucirumab (rt: 8,046 s) and R-vcMMAE (rt: 8,079). Column: TSKgel UP-SW3000 SEC column (2 µm, 4.6 mm x 300 mm, Tosoh Bioscience) Mobile phase: 100 mM sodium phosphate and NaCl (pH 6.8)

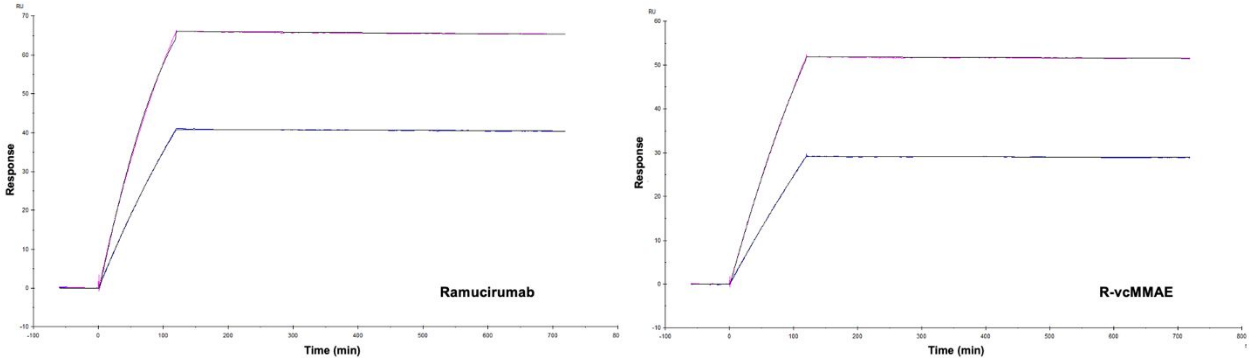

Figure S2 Surface plasmon resonance (SPR) sensorgrams of the reference antibody and the synthesized R-vcMMAE

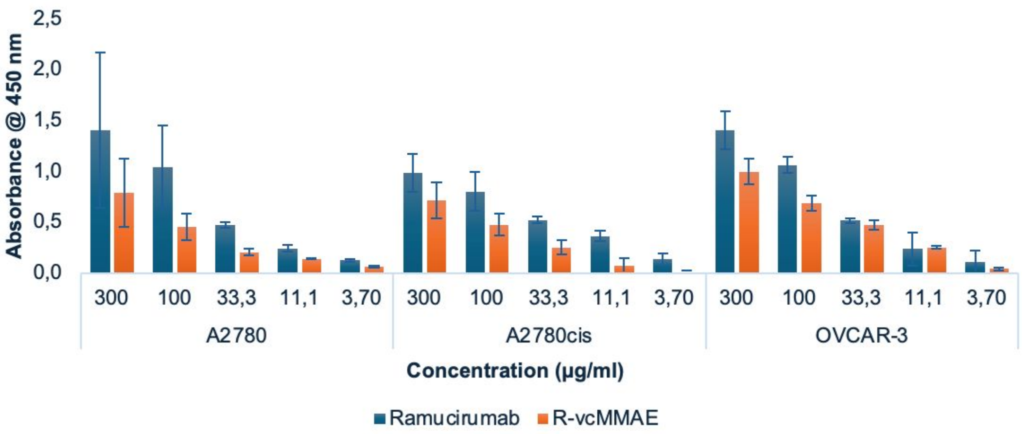

Figure S3 Cell-based ELISA results for Ramucirumab and R-vcMMAE on primary (A2780), cis-platin resistant primary (A2780cis) and metastatic (OVCAR-3) ovarian cancer cell lines. (Error bar showed SD of at least three independent experiments)

Table S1 Approximate IC$_{50}$ values (nM) of ramucirumab, R-vcMMAE, MMAE, and paclitaxel in ovarian cell lines (OSE-SV40, A2780, A2780cis, OVCAR-3), determined by cell viability assay. Values above 100 nM are indicated as ">100". ND: not detected or no measurable cytotoxicity within the tested concentration range or values above 1000 nM are indicated as ">1000".

Drug	OSE-SV40	A2780	A2780cis	OVCAR-3
**Ramucirumab**	ND	ND	ND	ND
**R-vcMMAE**	136.8	5.9	5.8	6.1
**MMAE**	2.9	0.12	0.13	1.4
**Paclitaxel**	>100	>100	>100	ND

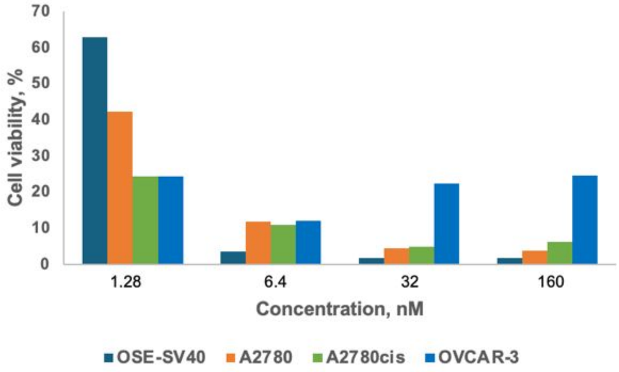

Figure S4 Cell viability analysis following treatment with a combination of unconjugated ramucirumab and free MMAE

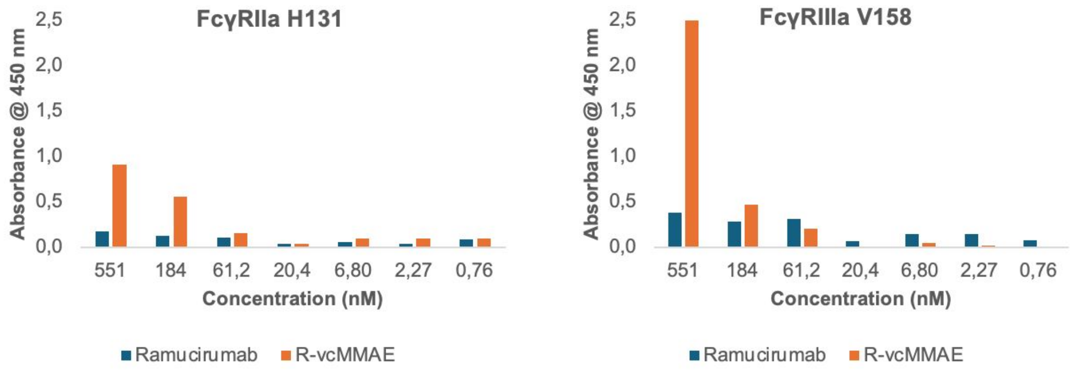

Figure S5 FcγRs binding profile of Ramucirumab and R-vcMMAE
